# Validation and evaluation of subject-specific finite element models of the pediatric knee

**DOI:** 10.1038/s41598-023-45408-5

**Published:** 2023-10-26

**Authors:** Ayda Karimi Dastgerdi, Amir Esrafilian, Christopher P. Carty, Azadeh Nasseri, Alireza Yahyaiee Bavil, Martina Barzan, Rami K. Korhonen, Ivan Astori, Wayne Hall, David John Saxby

**Affiliations:** 1 Griffith Centre of Biomedical and Rehabilitation Engineering (GCORE), Menzies Health Institute Queensland and the Advanced Design and Prototyping Technologies Institute (ADAPT), Griffith University, Gold Coast, QLD Australia; 2 Department of Technical Physics, University of Eastern Finland, Kuopio, Finland; 3 Department of Orthopedics, Children’s Health Queensland Hospital and Health Service, Brisbane, QLD Australia; 4 School of Engineering and Built Environment, Mechanical Engineering and Industrial Design, Griffith University, Gold Coast, QLD Australia

**Keywords:** Biomedical engineering, Cartilage

## Abstract

Finite element (FE) models have been widely used to investigate knee joint biomechanics. Most of these models have been developed to study adult knees, neglecting pediatric populations. In this study, an atlas-based approach was employed to develop subject-specific FE models of the knee for eight typically developing pediatric individuals. Initially, validation simulations were performed at four passive tibiofemoral joint (TFJ) flexion angles, and the resulting TFJ and patellofemoral joint (PFJ) kinematics were compared to corresponding patient-matched measurements derived from magnetic resonance imaging (MRI). A neuromusculoskeletal-(NMSK)-FE pipeline was then used to simulate knee biomechanics during stance phase of walking gait for each participant to evaluate model simulation of a common motor task. Validation simulations demonstrated minimal error and strong correlations between FE-predicted and MRI-measured TFJ and PFJ kinematics (ensemble average of root mean square errors < 5 mm for translations and < 4.1° for rotations). The FE-predicted kinematics were strongly correlated with published reports (ensemble average of Pearson's correlation coefficients (ρ) > 0.9 for translations and ρ > 0.8 for rotations), except for TFJ mediolateral translation and abduction/adduction rotation. For walking gait, NMSK-FE model-predicted knee kinematics, contact areas, and contact pressures were consistent with experimental reports from literature. The strong agreement between model predictions and experimental reports underscores the capability of sequentially linked NMSK-FE models to accurately predict pediatric knee kinematics, as well as complex contact pressure distributions across the TFJ articulations. These models hold promise as effective tools for parametric analyses, population-based clinical studies, and enhancing our understanding of various pediatric knee injury mechanisms. They also support intervention design and prediction of surgical outcomes in pediatric populations.

## Introduction

Knee biomechanics research is constrained by ethical considerations and demands extensive resources. Consequently, computational methods have been used for decades to simulate the mechanics of the human knee^1^. Among many computational approaches, finite element (FE) analysis has been the subject of considerable research focus and relied upon to study knee biomechanics at cell, tissue, and organ levels^2,3^. The computationally tractable alternatives to FE analysis, i.e., musculoskeletal (MSK) and neuromusculoskeletal (NMSK) models, offer insights into the knee joint kinematics, kinetics, and contact forces and in select cases surface pressures/stresses^4,5^, but are not able to calculate tissue- (and sub-tissue) level biomechanics, such as internal stress, strain, and fluid flow. This is notable as tissue level biomechanics are critical mechanobiological parameters regulating modelling (i.e., growth) and remodeling^6^. Moreover, FE analysis offers explicit control over boundary conditions, material properties, and structural alterations in parametric studies, which enables quantification of biomechanics such as contact forces/areas and stresses/strains and their distribution across all soft and hard tissue structures^7,8^.

Over the past three decades, numerous FE models of the knee have been developed to varying extents of complexity, accuracy, and functionality^1,9–12^. These models aimed to predict three-dimensional kinematics of tibiofemoral (TFJ) and patellofemoral (PFJ) joints in cadavers, healthy, and pathologic individuals^12–18^. However, there is a dearth of FE models and analyses focused on pediatrics populations, despite the clinical value of validated pediatrics FE knee models^19^. Given the increasing number of injuries and orthopedic interventions among youth^20^, validated FE models of the pediatric knee is of urgent need. A validated pediatric FE knee model would enable mechanistic study of the anatomy of the knee influences joint function in pathological conditions, but also support modifications (e.g., by removing constraints) to replicate unstable joint motion observed in pediatric cases, such as those caused by Anterior Cruciate Ligament (ACL) injuries or impaired contact in the lateral TFJ compartment^21^. However, such pediatric models are yet to be developed, and before they can be implemented with any confidence, they need to be validated and the performance under physiological conditions scrutinized.

Existing FE models of the knee have critical limitations reducing the confidence we may take in their simulations. Most of these models focus primarily on the isolated tibiofemoral joint, neglecting the interactions between patella and femur^19,22–26^. Additionally, some current models radically simplify the articular anatomy by neglecting articular cartilages^22,24–27^, menisci^4,19,24–27^, and other connective tissues^25,26,28–30^, as well as applying simplistic mechanical properties such as linear elasticity to tissue with known non-linear viscoelastic behavior^1,6,10^. To the best of our knowledge, there is only one FE study of the pediatric knee^19^, which underscores the need for development, validation, and evaluation of FE models of the pediatric knee.

The objectives of this study were to develop subject-specific FE models of the knee from eight healthy pediatric participants using an atlas-based approach to: (i) validate FE-driven kinematics of both TFJ and PFJ by comparing them to matched patient-specific in-vivo kinematics obtained from magnetic resonance imaging (MRI), and (ii) evaluate the kinematics generated by the validated FE models throughout the stance phase of the gait by comparing with experimental reports. For the former objective, passive FE simulations were performed at four different TFJ flexion angles, while for the latter, the patient-specific outputs of a NMSK pipeline were used as boundary conditions to the FE models. We contend the development, validation, evaluation of FE models of the pediatric knee would be a crucial step towards advancing FE modelling in the relatively underdeveloped field of pediatric knee biomechanics.

## Methods

### Overview of the workflow

**Figure 1:**
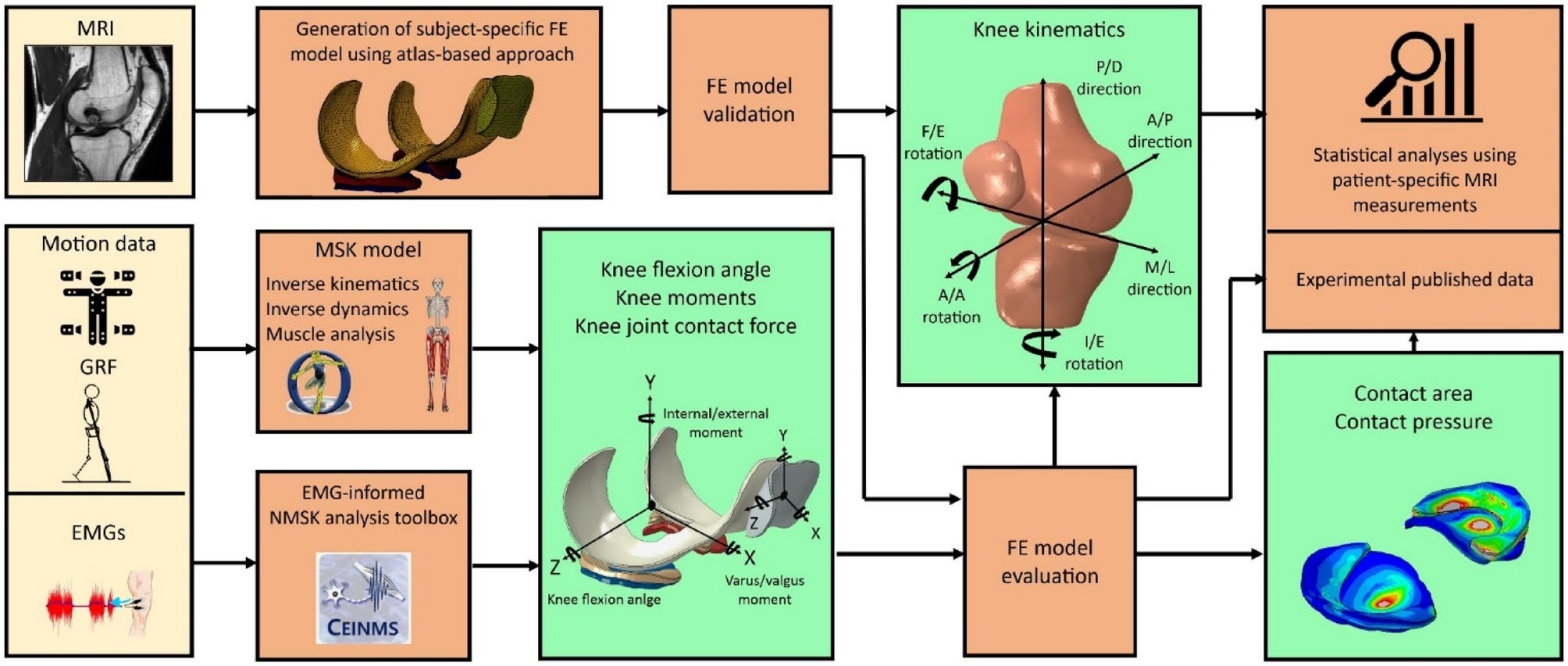
Overview of the workflow used in this study. Inputs, analyses, and outputs of the workflow are shown in yellow, orange, and green boxes, respectively. *EMG* electromyogram; *MRI* magnetic resonance imaging; *MSK* musculoskeletal; *NMSK* neuromusculoskeletal; *FE* finite element; *GRF* ground reaction forces; *F/E* flexion and extension; *P/D* proximal and distal; *A/P* anterior and posterior; *A/A* adduction and abduction; *I/E* internal and external; *M/L* medial and lateral.

A graphical overview of the study methods is depicted in Fig. 1. The study used an atlas-based technique to develop patient-specific FE models of eight pediatric knees, with each knee model based on the participant’s MRI. The FE models were then validated by performing passive simulations whereby model predicted kinematics were compared to corresponding measurements from previously collected MRI scans taken at four nominal TFJ flexion angles of 0°, 7°, 15°, and 25°^21^. Subsequently, a sequentially linked NMSK-FE pipeline was used to simulate knee kinematics, contact areas, and contact pressures developed during the stance phase of walking gait. These gait biomechanics were then evaluated by comparing with previously published experimental reports.

### Participants, medical imaging, biomechanical data acquisition, and preprocessing

Previously collected data from eight typically developing children and adolescents were used to this study (i.e., TD1–TD8). This cohort was comprised of four males and four females, with average age 14.0 ± 2.6 years, mass 51.1 ± 10.5 kg, and height 1.64 ± 0.11 m. Ethical approval was granted by the Human Research Ethics Committee of Children's Health Queensland Hospital and Health Services (HREC/13/QRCH/197). Written informed consent was obtained from guardians of each participant prior to any testing and all the experiments were performed in accordance with relevant guidelines and regulations (principles set by the Declaration of Helsinki).

A regional scan of each participant’s unloaded right knee was performed using a magnetic resonance imaging (MRI) scanner (Siemens MAGNETOM Skyra 3T scanner, Germany). The scan used a dedicated knee coil, employed a 3D SPC T_2_ imaging technique with 0.53 mm slice thickness, and had a 0.53 × 0.53 × 0.53 mm3 voxel size. The knee’s TFJ was posed to ~ 0° flexion during the scan.

Following MRI, each participant underwent gait analysis at Queensland Children’s Motion Analysis Service (QCMAS) within the Centre for Children’s Health Research (Brisbane, Australia). Therein, each participant was given instructions to perform a series of walking trials at their self-selected speed. To track the motion of their body segments, retroreflective markers were placed atop specific anatomic landmarks on the skin surface^31^, while an optical motion capture system, consisting of ten cameras (Vicon Motion Systems Ltd, Oxford, UK), acquired whole-body motion (sampling at 100 Hz). From each participant’s right leg, surface electromyography (EMG) electrodes were applied atop 10 muscles: gluteus maximus, semitendinosus, biceps femoris long head, rectus femoris, vastus medialis and lateralis, gastrocnemius, gracilis, tensor fasciae latae, and sartorius. Electrode placement was consistent with guidelines outlined in Surface Electromyography for the Non-invasive Assessment of Muscles^32^. The EMG were recorded using a wireless surface system (Noraxon, Scottsdale, AZ, USA) sampling at 2400 Hz. Four ground-embedded force platforms (510 mm × 465 mm, AMTI, Watertown, MA, USA) were employed to measure ground reaction forces, sampling at 1000 Hz.

Motion and ground reaction force data were filtered using a 4th-order (2nd-order cascaded once to remove phase effects) low-pass Butterworth filter with a  nominal cut-off frequency of 6 Hz. The EMG underwent bandpass filtering (range 30–300 Hz), followed by full wave rectification, and finally low-pass filtering (cut-off 6 Hz) to generate linear envelopes. Each linear envelope was then amplitude-normalized to its respective maximum value identified from all recorded trials.

### Neuromusculoskeletal pipeline

In the NMSK pipeline, two stages of modelling were undertaken to generate boundary conditions for the FE models. First, the external biomechanics were modelled and then muscle dynamics computed. For the external biomechanics, the Rajagopal 2015 MSK model was employed^33^ within the OpenSim modelling environment (Version 3.3, Stanford University, CA, USA)^34^. This model had a total of 37 degrees of freedom and 80 muscle–tendon unit actuators (MTU). The model was adjusted to the mass, inertia, and segmental dimensions of each participant based on their body mass and the position of specific bony landmarks and hip joint centers^35^. To ensure the preservation of dimensionless muscle fiber and tendon operating ranges, optimal fiber and tendon slack lengths of the MTU from the lower limbs were optimized subsequent to scaling (above)^36^. The maximum isometric strength of each muscle was then updated using the Handsfield equations based upon the participant’s mass and height^37^. Following the personalization of the model, generalized coordinates (i.e., motions), generalized loads (i.e., net joint forces and moments), and MTU kinematics (i.e., lengths, moment arms and lines of action) were solved using inverse kinematics (IK), inverse dynamics (ID) and muscle analysis (including line-of-action plug-in), respectively.

The second stage of the NMSK pipeline involved determining lower-limb muscle dynamics and joint contact forces. To this end, ID, IK, and MTU modelled in OpenSim were used, along with muscle activation patterns (i.e., normalized linear envelopes), to estimate muscle dynamics using the Calibrated EMG-Informed Neuromusculoskeletal Modelling Toolbox (CEINMS)^38^. The CEINMS was used first in calibration mode, to refine muscle model parameters to best fit each participant’s experimental gait data. This was done by using three arbitrarily selected walking trials, where parameters governing muscle activation dynamics, maximum isometric forces, and optimal fiber and tendon slack lengths were adjusted (within physiological boundaries) such the model generated muscle torques which matched those from ID in the sagittal plane about hip, knee, and ankle joints of the right limb (i.e., EMG instrumented).

Once calibrated, CEINMS was run in the EMG-assisted mode due to this neural mode’s ability to strike a favorable balance between generating joint torques that well-match those from ID and effectively incorporating experimentally measured muscle excitation (i.e., EMG linear envelopes)^39^ (Supplementary material Figures S1 and S2). The experimentally measured EMG from the ten lower limb muscles, MTU kinematics, and joint torques from IK were used to drive CEINMS in EMG-assisted mode, which minimally adjust experimentally collected EMG and synthesize excitations for the remaining 30 MTU in the lower limb. Biomechanical outputs from both OpenSim and CEINMS included model motions, net joint torques, as well as lower limb muscle forces, tibiofemoral contact forces, and patellofemoral contact forces. These outputs were subsequently used in the FE pipeline as boundary conditions.

### Finite element pipeline

#### Geometry and material properties

To develop patient-specific geometries for FE analysis, an atlas-based approach was used^40^. This approach begins with a template FE model of the knee, which was then anisotropically scaled based on subject-specific joint morphology measured from MRI. To this end, sagittal and frontal plane image slices that displayed the maximum anteroposterior length of the medial and lateral femoral condyles were selected. Subsequently, various dimensions of cartilage and menisci were measured separately for both the medial and lateral sides (see Figure S3). These measurements served as the basis for scaling the thickness and length of various anatomical structures, including the femur, tibia, patella, and menisci. The scaling factors were determined using average values to maintain realistic contact surfaces and stress distributions. Ligament insertion points were also scaled based on anatomical data. To create subject-specific FE models, an in-house MATLAB script was used, ensuring the nodal coordinates of each part in the Abaqus input file (template model) were appropriately scaled. This streamlined approach facilitated generation of subject-specific FE models while maintaining consistency across other elements of the model. The template model consisted of femoral, patellar, and tibial cartilages, menisci, and major knee ligaments. As the deformation of bones in response to applied loads is negligible compared to that of soft tissues^41^, bones were considered rigid and excluded from FE analysis^40^.

Knee ligaments, including ACL, posterior cruciate ligament (PCL), lateral collateral ligament (LCL), medial collateral ligament (MCL), lateral patellofemoral ligament (LPFL), medial patellofemoral ligament (MPFL), and patellar ligament, were represented in the template model as bundles of nonlinear springs and their respective insertion points were extracted from each participant’s MRI. Ligament pre-strain and stiffness were set according to a previously validated adult model (Table 1)^4,42,43^. Cartilages and menisci geometries were obtained from the atlas-based approach.

**Table 1:** Material parameters of anterior cruciate ligament, posterior cruciate ligament, lateral collateral ligament, and medial collateral ligament used in this study^42^.

Ligament	Bundle	K(kN)	Reference strain
Anterior cruciate ligament	aAC	5	0.06
	pAC	5	0.10
Posterior cruciate ligament	aPC	3	− 0.24
	pPC	3	− 0.12
lateral collateral ligament	aLC	2	0.038
	sLC	2	0.038
	pLC	2	0.08
Medial collateral ligament	aMC	1.83	0.04
	iMC	1.83	0.04
	pMC	1.83	0.057

Within the majority of FE models in the literature, as in our study, several knee tissues restraining the joint (e.g., knee capsule, skin, remaining shank, and thigh masses, etc.) are excluded. Consequently, FE models often cannot achieve convergence without implementing the effects of those excluded tissue. To converge the FE models, previous studies have either applied an approximate fraction (~ 10–50%) of the magnitude of knee moments and contact forces^12,44^, or disregarded some degree of freedom (DoF) or boundary conditions (e.g., patellar moments)^41,45^. Adopted from previous studies which used multi-DoF knee models^4,43^, we modulated secondary knee DoF stiffness to account for the knee tissues neglected in our FE model, rather than reducing the magnitude of the applied loading. The stiffnesses of these elements were adjusted to facilitate model convergence and they remained constant throughout validation and evaluation simulations and across participants (Table 2). The attachments of the menisci horns were modelled as spring bundles, with a total stiffness of 336 N/mm and 381 N/mm for the anterior and posterior sides, respectively^46^. Femoral, tibial, and patellar cartilages were modelled as depth-dependent transversely isotropic poroelastic materials, while menisci were modelled as transversely isotropic elastic material (Table 3)^47^.

**Table 2:** Added stiffnesses to the secondary knee degrees of freedom, accounting for the elements excluded from the finite element model (e.g., knee capsule, skin, limb mass, etc.). Added stiffnesses remained consistent for all the subjects (i.e., TD1-8) in both validation and evaluation simulations.

Degree of freedom	Femur	Patella-femur
Anteroposterior	100 N/mm	–
Internal/external	425 N·mm/deg	425 N·mm/deg
Abduction/adduction	–	425 N·mm/deg

**Table 3:** Material parameters for the knee joint cartilages and menisci used in this study^47^.

Material parameter	Cartilage			Menisci
	Superficial zone	Middle zone	Deep zone	
${E}_{p}$(MPa)	24	16.97	8.49	159.6
${E}_{t}$(MPa)	0.46	0.46	0.46	20
${v}_{p}$	0.42	0.42	0.42	0.3
${v}_{tp}$	0.06	0.08	0.12	0.01
${G}_{t}$	12	8.45	4.24	8
$K$	1	1	1	–
${e}_{0}$	4	4	4	–

${E}_{p}$: in-plane Young's modulus, ${E}_{t}$: out-of-plane Young's modulus, ${v}_{p}$: in-plane Poisson's ratio, ${v}_{tp}$: out-of-plane Poisson's ratio, ${G}_{t}$: out-of-plane shear modulus, $K$ : permeability, and ${e}_{0}$: void ratio.

### Loading and boundary conditions

#### Validation

For all FE models developed in this study, the inferior surface of the tibial cartilages was restricted in all directions (i.e., encastre constraint used in Abaqus). Two reference points for femur and patella were defined to apply inputs to the FE models. Nodes on the femoral cartilage-subchondral bone and patellar cartilage-subchondral bone interfaces were coupled to femoral and patellar reference points, respectively^12^.

To validate the FE models, passive simulations were conducted using Abaqus/Standard soils consolidation solver, separately for each participant (i.e., TD1-TD8). Inputs to the models consisted of TFJ flexion angles of 0°, 7°, 15°, and 25° and a superior/inferior displacement of 3 mm to establish initial contact between model components. The remaining model DoF were free to move in response to applied loads. In validation simulations, femur had 4 active DoF, consisting of 2 translations (anteroposterior and mediolateral) and 2 rotations (abduction/adduction and internal/external), whereas patella had 6 active DoF, consisting of 3 translations (anteroposterior, mediolateral, and proximal–distal) and 3 rotations (abduction/adduction, internal/external, and flexion/extension).

#### Evaluation

To evaluate the FE models, kinematics, and kinetics from the NMSK simulations of gait were applied to femoral and patellar reference points according to the previous studies^12,40^ (see Figure S4 in Supplementary materials). The TFJ flexion angle (from IK analysis), knee internal/external and varus/valgus moments (i.e., from ID in addition to moments generated by muscles in abduction/adduction and internal/external DoF), and tibiofemoral contact forces (in three directions of XYZ) were applied to the femoral reference point. While explicit representation of estimated muscle forces was absent in the FE models, their effects were accounted for by incorporating the moments they generated into the varus/valgus and internal/external moments obtained through ID. In addition, muscles contributions were implicitly integrated through the joint contact forces estimated by CEINMS. Similarly for the patella, patellar flexion/extension, abduction/adduction, and internal/external moments generated by the quadriceps and the patellofemoral contact force (in three directions of XYZ) were applied to the patellar reference point (see Figure S4 in Supplementary materials). The femur possesses 5 active degrees of freedom (DoF), consisting of 3 translations (anteroposterior, mediolateral, and distal–proximal) and 2 rotations (abduction/adduction and internal/external). The patella has 6 active DoF, including 3 translations (anteroposterior, mediolateral, and proximal–distal) and 3 rotations (abduction/adduction, internal/external, and flexion/extension). Finally, the stance phases for ~ 3–5 walking trials per participant were simulated using Abaqus/Standard soils consolidation solver.

#### Data analysis and statistics

For validation simulations, root mean square error (RMSE) was computed for each participant between their knee (TFJ and PFJ) kinematics predicted by FE model and those measured directly from MRI^21^. The RMSE was computed across the four discrete TFJ flexion angles and averaged. Additionally, the similarities of the TFJ and PFJ kinematic curves predicted by FE models and those from published reports^48–50^ were examined using Pearson’s correlations.

For the simulations of walking gait, TFJ and PFJ kinematics, TFJ contact areas, and TFJ contact pressures during stance phase were compared with experimental reports to ensure physiological predictions^51–56^. Within the TFJ, four contact regions consisted of (1) femoral cartilage to medial tibial cartilage, (2) medial meniscus to medial tibial cartilage, (3) femoral cartilage to lateral tibial cartilage, and (4) lateral meniscus to lateral tibial cartilage. Finally, total contact area and contact area ratio were calculated using area of the four contact regions.

## Results

### Validation

#### Knee joint kinematics

**Figure 2:**
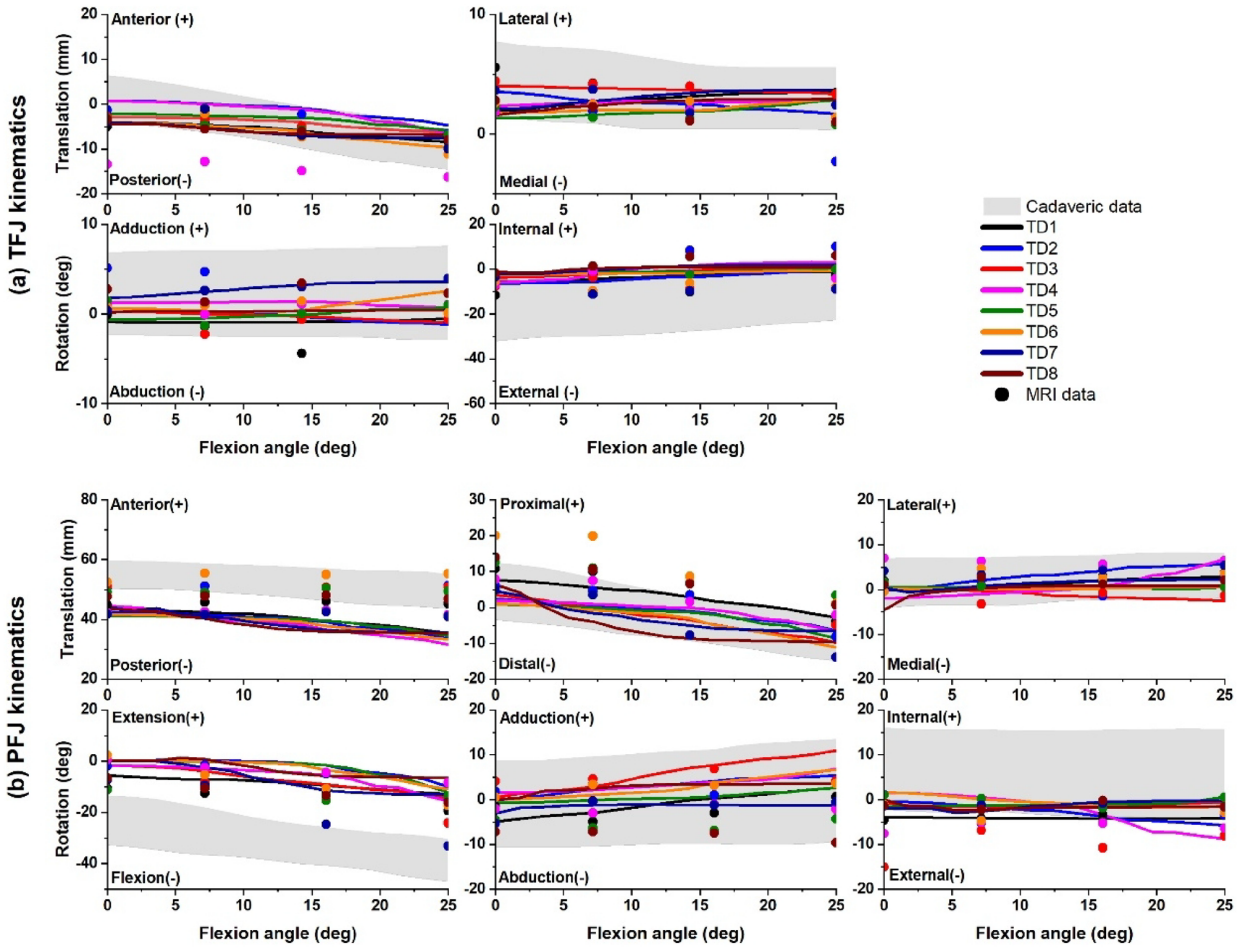
Comparison of kinematics from (**a**) tibiofemoral joint (TFJ) and (**b**) patellofemoral joint (PFJ) as simulated by the FE models (colored lines), corresponding MRI-based measurements (dots)^21^, and published values from measurements taken from cadaveric specimens^48–50^ (grey) across a range of TFJ flexion angles.

The FE models well replicated MRI-based measurements of the same subjects^21^ of both TFJ and PFJ joint kinematics. The RMSE between model prediction and MRI-based measurements for all DoF at TFJ and PFJ were generally acceptable across (n = 8) participants. Modelled TFJ had ensemble average RMSE < 2.82 mm for anteroposterior and mediolateral translations, < 1.63° for abduction/adduction rotation, indicating generally good agreement with MRI-based measures (Table 4). The TFJ internal/external rotation (Fig. 2), had higher average RMSE (~ 5.24°, indicating divergence with MRI-based measurements). When compared with MRI-based measurements of PFJ kinematics, FE model predictions had ensemble average RMSE for rotations < 4.49° and translations < 6.98 mm (Table 4).

**Table 4:** Root mean square error and Pearson’s correlation coefficients (ρ), along with their average and standard deviation, are presented.

Joint	Rotation (°)			Translation (mm)		
	F/E	A/A	I/E	A/P	P/D	M/L
RMSE between model and MRI-based measurements of joint kinematics						
TFJ	–	1.63 (0.95)	5.24 (1.9)	2.82 (3.80)	–	1.31 (0.55)
PFJ	7.12(3.14)	3.21 (1.47)	3.13 (2.89)	9.85 (3.89)	8.29 (4.6)	2.80 (1.55)
Pearson’s correlation coefficients between model and cadaver-based measurements						
TFJ	–	0.41 (0.13)	0.93 (0.073)	0.96 (0.02)	–	− 0.67 (0.58)
PFJ	0.96(0.03)	0.89 (0.16)	0.62 (0.41)	0.96 (0.02)	0.95 (0.03)	0.86 (0.13)

The root mean square error quantifies the error between knee kinematics measured from magnetic resonance imaging^21^ and the corresponding values predicted by the finite element models. The Pearson's correlation coefficients quantify to correlation between knee kinematics measured from cadavers as reported in the literature^48–50^ and corresponding kinematics predicted by the finite element models.

*F/E* flexion and extension; *A/A* adduction and abduction; *I/E* internal and external rotation; *A/P* anteroposterior; *P/D* proximodistal; *M/L* mediolateral; *TFJ* tibiofemoral joint; *PFJ* patellofemoral joint; *RMSE* root mean square error.

The FE models also predicted TFJ anteroposterior translation and internal/external rotation which were strongly positively correlated (ρ > 0.9) with published reports^48–50^ (Table 4). Model predicted TFJ mediolateral translation and abduction/adduction rotation showed weaker and highly variable correlations with published reports (range: − 0.67 < ρ < 0.41) (Table 4). Model predicted PFJ kinematics displayed generally strong positive correlation with published reports of PFJ translations and rotations, except for internal/external rotation which was only moderately correlated (ρ ≥ 0.62) (Table 4). Overall, model predicted TFJ and PFJ kinematics generally well tracked corresponding kinematics measured from cadaveric specimens (Fig. 2).

### Evaluation

#### Knee joint kinematics

**Figure 3:**
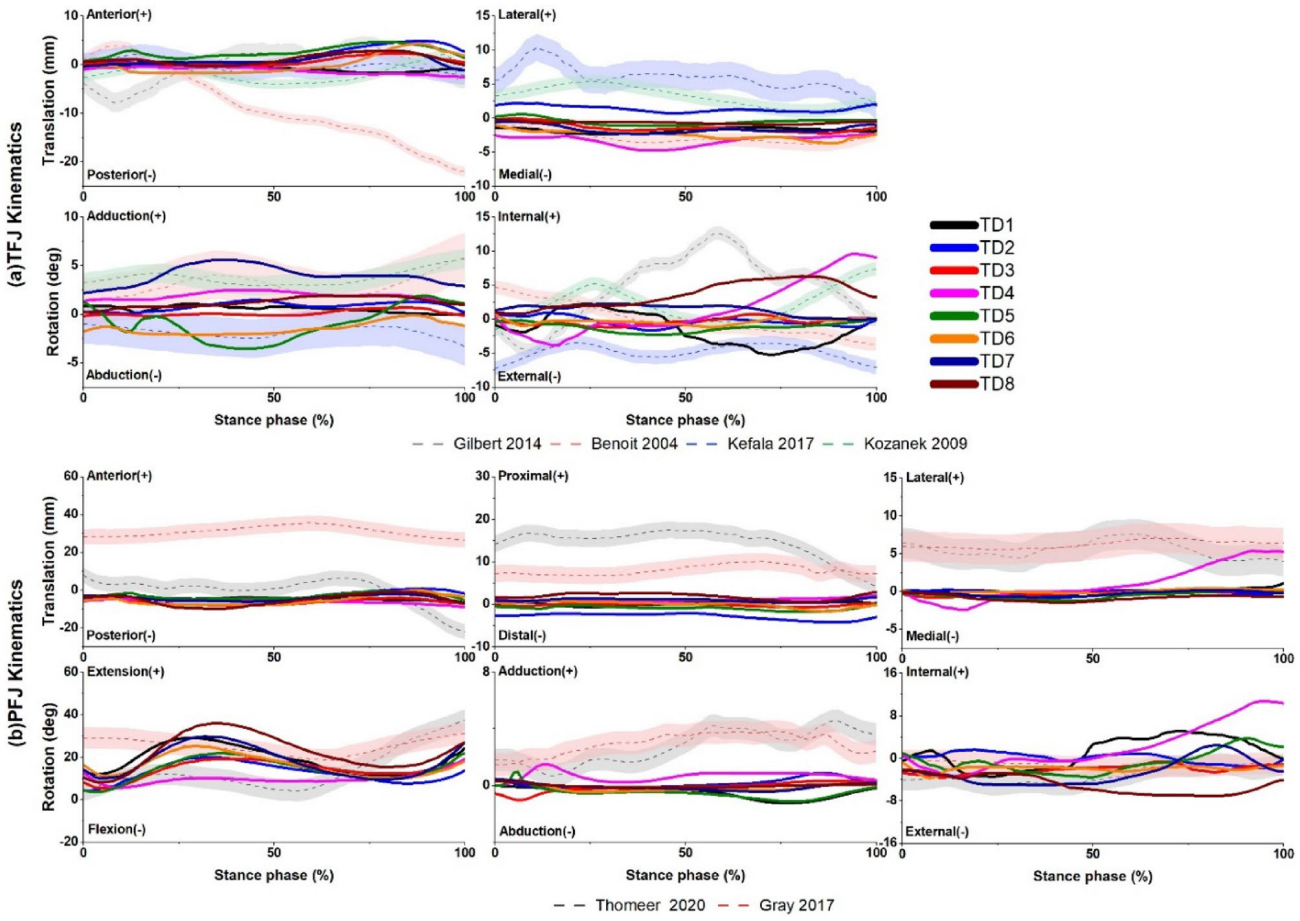
Comparison between kinematics from (**a**) tibiofemoral joint (TFJ) and (**b**) patellofemoral (PFJ) simulated from the NMSK-FE models used in this study and those measurements from experiments^51–54^ during stance phase of walking gait.

The TFJ and PFJ kinematics in published reports^51–54^ have substantial variations in their patterns and magnitudes (Fig. 3). Despite this variability, knee kinematics simulated by the NMSK-FE models were comparable with the bulk of these values reported in the literature. However, there were differences in magnitudes between literature and simulated values for both translations and rotations, particularly for PFJ kinematics.

#### Contact area

**Figure 4:**
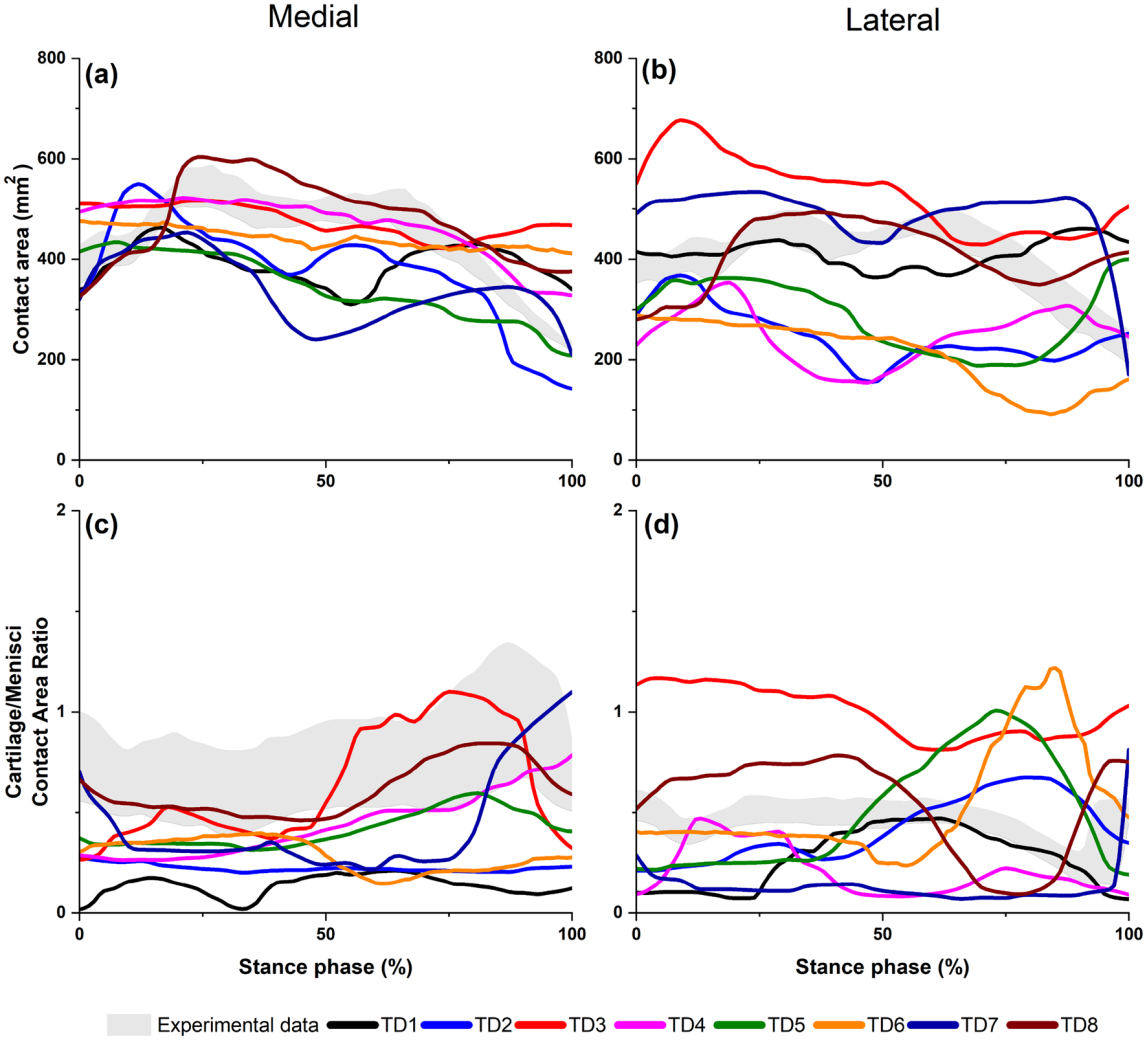
The contact area of NMSK-FE models across stance phase of walking gait compared to published data^52^; (**a**) and (**b**) total medial and lateral contact areas respectively; (**c**) and (**d**) ratio of cartilage to cartilage and menisci to cartilage contact area ratio on the medial and lateral tibial plateau, respectively.

Figure 4 illustrates the distribution of contact areas between menisci and tibial cartilages throughout stance phase of walking gait for all NMSK-FE models. Results showed contact area under the medial meniscus was larger than cartilage-cartilage contact area within the medial tibial plateau. The opposite was observed for a few participants in the lateral tibial plateau, specifically at pre-swing phase of stance. Overall, NMSK-FE models simulated contact areas for both medial and lateral tibial cartilages comparable to those measured in-situ^52^. The ratios of medial cartilage-to-cartilage to menisci contact area from in-situ measurements and NMSK-FE models were 0.74 ± 0.1 and 0.39 ± 0.16, respectively (Fig. 4c). The ratio of lateral cartilage-to-cartilage to menisci contact area by NMSK-FE models was 0.44 ± 0.25, while in-situ measurements yielded 0.46 ± 0.067 (Fig. 4d).

#### Cartilage mechanical response

**Figure 5:**
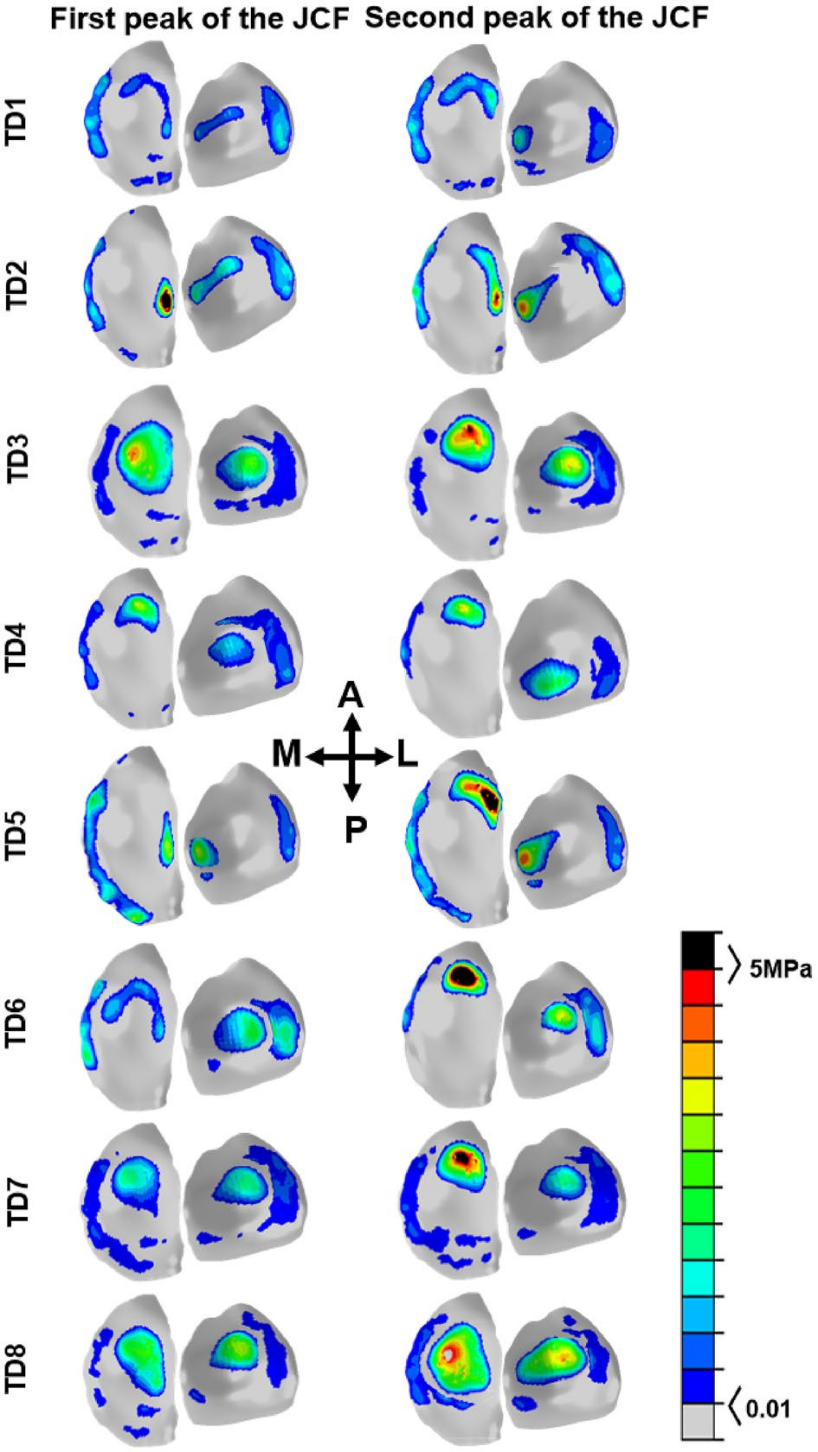
Contact pressure during the first and second peaks of JCF estimated by NMSK-FE models of this study. Stresses less than 0.01 are illustrated in grey, while stresses above 5 MPa are highlighted in black.

The magnitude and distribution of contact pressures simulated by NMSK-FE models are shown in Fig. 5. Generally, contact pressure induced by the second peak of tibiofemoral contact force was of greater magnitude than that generated by the first peak of tibiofemoral contact force. The cartilage–cartilage zone bore a greater contact stress than the menisci-cartilage zone during both peaks of tibiofemoral contact forces for both medial and lateral tibial plateaus. Compared to the experimental values of lateral contact pressure^52^, NMSK-FE models predicted lower magnitude contact pressures on the lateral tibial cartilage.

## Discussion

In this study, we used an atlas-based approach to develop subject-specific FE models of the knee joint in eight typically developing pediatric individuals. Validation simulations showed FE models well predicted both TFJ and PFJ kinematics compared to corresponding measurements of the same pediatric individuals made using MRI^21^. Following validation, subject-specific NMSK-FE models were used to simulate the stance phase of walking gait. The resulting knee kinematics, contact areas, and contact pressures were consistent with prior experimental reports^51–54^. This study demonstrated the atlas-based FE models can be sequentially linked with NMSK models to simulate pediatric TFJ and PFJ biomechanics, and these linked-models account for the complex interaction between muscle, ligament, and articulating surfaces, rather than relying on assumptions derived solely from studies involving adult cadaveric samples or clinical observations.

### Validation results

Compared with MRI-based measurement^21^, FE models simulated the passive kinematics of both TFJ and PFJ with minimal deviation for most DoF. Internal/external rotation of the TFJ had the largest error of TFJ DOF (i.e., 5.24°), likely due to the pronounced external rotation present in the pediatric TFJ, as observed in previous studies^21^. The pediatric TFJ experiences this external rotation partly because of the joint’s laxity when fully extended and in the supine position (as in the MRI scanner). In this study, the FE model ligament properties were not subject-specific and pre-strain values were taken from adult models, which might restrict the initial internal/external rotation of the pediatric knee models compared to their real anatomical counterparts. Moreover, our use of stiffness support to TFJ and PFJ internal/external rotations in the FE models to assist model convergence likely also limit initial internal/external rotation of the TFJ. Despite these considerations, the modelled TFJ internal/external rotation in pediatric participants displayed a strong correlation (ρ > 0.9) with published reports, indicating similar patterns when compared to reports on adult cadaveric specimens^48–50^.

Compared with prior reports in the literature, passive TFJ and PFJ kinematics predicted by the FE model showed strong correlations with knee kinematics measured during experiments conducted on cadaveric specimens for most DoF^48–50^. Of notable exception, FE model predictions of TFJ mediolateral translations and abduction/adduction rotations showed weak and variable correlations (range: − 0.67 < ρ < 0.41) with published reports (Table 4), although they generally overlapped with the range of motion of the TFJ and PFJ kinematics reported in the literature. The discord between simulation and cadaveric data for mediolateral translation can be attributed to variations in the orientation of the TFJ within FE models and those of the published data. The orientation of the TFJ has a notable impact on the trajectory of movement in the mediolateral direction of FE models once contact is established. This effect is particularly pronounced during passive simulation when the direction of joint movement is not determined by external forces (e.g., muscle). Moreover, it has been demonstrated the abduction/adduction rotation of the TFJ is highly dependent on the geometric characteristics and orientation of the TFJ joint^53^. Therefore, the disparity between FE simulations and cadaveric data regarding abduction/adduction rotation may arise from the geometrical differences in the knee joint in adult and pediatric individuals.

Across the 8 pediatric participants, their FE models showed acceptable prediction accuracy compared with corresponding MRI-based measurements, but within-subject variations in kinematic predictions were observed (i.e., TD1-8)^21^. This variability indicates subject specificity matters to model performance, hence the unequal performance across participants. Indeed, the FE models developed in this study were not fully subject-specific, e.g., they did not incorporate the mechanical properties and specifications of articular constituents and ligaments. For the models that performed best, their model ligament properties may have been good approximation of their subject-specific values, and in cases of poor model performance perhaps less so. Overall, average kinematics predicted by FE models closely corresponded to both MRI-based measures^21^ and published reports^48–50^. This indicates our models well capture the typical kinematics of the TFJ and PFJ, even in the absence of subject-specific tissue mechanical parameters.

### Evaluation results

#### Kinematics

During walking gait, experimental studies reported widely varying magnitudes and patterns for TFJ and PFJ kinematics^51–54^. This can be attributed to real variability between individuals and motor tasks, and due to differences in measurement methods. However, the FE model predictions of TFJ and PFJ kinematics were consistent with reports from experimental studies^51–56^. Specifically, model predicted TFJ kinematics had a similar range as those measured in experiments^51–54^. Model predicted PFJ kinematics had less agreement with the magnitudes reported in the literature^55,56^. Comparisons between model predictions and published literature must always be tempered by acknowledging the kinematics are from different people/specimens and of different maturities (i.e., adults vs. children), which ultimately results in variations in muscle forces, external joint moments, knee orientations, gait coordination, and ligament pre-strains, all of which are subject-specific. Furthermore, the smaller geometries of pediatric knees compared to adult knees can contribute to lower magnitudes of translations during stance phase of gait, as translations are reported in parametric form and not normalized to the size of the participant’s joint. The variability in kinematics across the FE models (i.e., TD 1–8) could be indicative of the role of both model geometry and boundary conditions from NMSK simulations. Indeed, the workflow in this study included variations in gait style, speed, knee orientation, and others across the subjects.

#### Contact area

The ratio of contact areas between cartilage-to-cartilage and menisci-to-cartilage predicted by the NMSK-FE models showed minimal deviation from published reports^52^, particularly in the medial tibial compartment. During the first peak in TFJ contact force, contact on the medial plateau was evenly distributed between cartilage-to-cartilage and meniscus-to-cartilage. However, during the second peak in TFJ contact force in the latter half of stance, the contact ratio shifted towards cartilage-to-cartilage, consistent with experimental and numerical reports^12,52^. This suggests the role of the medial meniscus as a load bearing structure is primarily during early stance, and this role diminishes as gait progresses. In the lateral compartment, the ratio of contact areas between cartilage-to-cartilage and meniscus-to-cartilage was unequal throughout gait for models TD3, TD5, and TD8. This suggests the lateral meniscus was not the primary structure for contact distribution but was particularly notable during pre-swing phase of stance. However, experimental study contravenes this result^52^. Interestingly, models for TD3 and TD8 exhibit similar patterns to the literature^52^. This discrepancy may be attributed to differences in geometry and knee orientation across models, which can impact the contact between menisci and cartilages. Furthermore, the experimental study^52^ did not incorporate the varus-valgus moment^52^, which is indeed present during gait and was included in our FE models. This disparity could result in increased loads and contact on the lateral side of the FE models compared to the experimental conditions.

### Limitations

This study had several limitations that should be considered. First, an atlas-based approach was employed for the development of FE models using an adult-knee template^40^, which, while efficient and accurate, does not yield exactly the same surface geometries as a manually segmented model. The creation of fully subject-specific models from imaging, without templates as intermediaries, is a time-consuming and intricate process encompassing image segmentation, mesh generation, model assembly, and the attainment of a converged solution (e.g., 4–16 weeks). Consequently, this resource-intensive procedure limits the application of these models to a very small number of participants for analysis. Use of an atlas-based method enables rapid and practical modelling, rendering it well-suited for large cohorts, including both clinical and research purposes. Second, the ligaments in the FE model were represented as spring elements rather than deformable 3D representations that can be used to compute spatial variations in strain. However, it has been reported the effect of this simplification of the ligament representation on knee kinematics is minimal^57^. Third, the mechanical properties of the knee tissues (i.e., cartilages, menisci, and ligaments) were not subject-specific. Viable (i.e., accurate, accessible, and non-invasive) methods for obtaining subject-specific material properties of the knee’s soft tissues (e.g., cartilage and menisci) are currently unavailable, nor it is clear the impact personalized soft-tissue properties would have on model simulations investigated in this study. Consequently, soft tissue material parameters used in this study were adopted from existing literature data based on experiments conducted on adult specimens. Forth, the validation with MRI-measured kinematics was limited to small TFJ flexion angles due to physical confines of the MRI bore diameter. Last, the lack of published kinematic data for pediatric knees necessitated the comparison of NMSK-FE simulations with experimental data collected from adults. This assumed comparable knee kinematics between the two populations, which we know may not be appropriate.

### Application and future development

In the context of the reported errors, development, and validation of FE models of the pediatric knee which mimic *in-vivo* biomechanics may have significant clinical implications in the future. First, these models can augment surgical interventions by providing tools to simulate implant stresses and thereby reduce the risk of implant failure. Further, the models can be used to simulate ligament mechanics following reconstruction to examine the risk of secondary rupture and other post-surgical complications. Third, these models can be used to evaluate the biomechanics of the knee in various pathological conditions, including patellar dislocation, malalignment, and cerebral palsy. They can also be readily adjusted to simulate an unstable pediatric knee, as seen in cases of ACL deficiency or loss of contact in the lateral tibiofemoral compartment. Last, these models can provide insights into post-treatment joint functionality, such as following ACL reconstruction, as this procedure may contribute to tissue degeneration and the early onset of knee osteoarthritis. Specifically, our forthcoming study aims to use these validated FE models to optimize parameters related to ACL reconstruction surgery in pediatric populations, such as graft size, type, location, and pretension.

## Conclusion

In this study, we developed FE models of the pediatric knee that tracked corresponding in-vivo measured kinematics. The combination of atlas-based FE models with a subject-specific NMSK modelling enabled the prediction of kinematics, kinetics, and tissue-level mechanics of both TFJ and PFJ during physiological conditions (e.g., walking) consistent with experimental published data. This model offers the potential to augment the analysis of motor tasks and rehabilitation activities, as well as predict biomechanical outcomes of different pediatric knee surgeries, such as knee osteotomy and ACL reconstruction.

## Competing Interests

The authors declare no competing interests.

### Supplementary Information

Supplementary Information.

## Data Availability

The datasets generated during and/or analyzed during the current study are available from the corresponding author on reasonable request.

## References

[1] Cooper RJ, Wilcox RK, Jones AC (2019). Finite element models of the tibiofemoral joint: A review of validation approaches and modelling challenges. Med. Eng. Phys..

[2] Erdemir A, Besier TF, Halloran JP, Imhauser CW, Laz PJ, Morrison TM, Shelburne KB (2019). Deciphering the “art” in modeling and simulation of the knee joint: Overall strategy. J. Biomech. Eng..

[3] Kazemi, M., Dabiri, Y. & Li, L. Recent advances in computational mechanics of the human knee joint. *Comput. Math. Methods Med.* **2013** (2013). 10.1155/2013/718423 PMC3590578 23509602

[4] Lenhart RL, Kaiser J, Smith CR, Thelen DG (2015). Prediction and validation of load-dependent behavior of the tibiofemoral and patellofemoral joints during movement. Ann. Biomed. Eng..

[5] Serrancolí, G., Torner, J., Perelli, S. & Monllau, J. C. On the use of mesh-based joint contact models within simulations using automatic differentiation, pp. 244–249.

[6] Mukherjee S, Nazemi M, Jonkers I, Geris L (2020). Use of computational modeling to study joint degeneration: A review. Front. bioeng. biotechnol..

[7] Erdemir A, Guess TM, Halloran J, Tadepalli SC, Morrison TM (2012). Considerations for reporting finite element analysis studies in biomechanics. J. Biomech..

[8] Taylor M, Prendergast PJ (2015). Four decades of finite element analysis of orthopaedic devices: Where are we now and what are the opportunities?. J. Biomech..

[9] Halonen K, Dzialo CM, Mannisi M, Venäläinen M, de Zee M, Andersen MS (2017). Workflow assessing the effect of gait alterations on stresses in the medial tibial cartilage-combined musculoskeletal modelling and finite element analysis. Sci. Rep..

[10] Kiapour A, Kiapour AM, Kaul V, Quatman CE, Wordeman SC, Hewett TE, Demetropoulos CK, Goel VK (2014). Finite element model of the knee for investigation of injury mechanisms: Development and validation. J. Biomech. Eng..

[11] Lenhart RL, Kaiser J, Smith CR, Thelen DG (2015). Prediction and validation of load-dependent behavior of the tibiofemoral and patellofemoral joints during movement. Ann. Biomed. Eng..

[12] Esrafilian A, Stenroth L, Mononen ME, Tanska P, Van Rossom S, Lloyd DG, Jonkers I, Korhonen RK (2020). 12 degrees of freedom muscle force driven fibril-reinforced poroviscoelastic finite element model of the knee joint. IEEE Trans. Neural Syst. Rehabil. Eng..

[13] Ali AA, Shalhoub SS, Cyr AJ, Fitzpatrick CK, Maletsky LP, Rullkoetter PJ, Shelburne KB (2016). Validation of predicted patellofemoral mechanics in a finite element model of the healthy and cruciate-deficient knee. J. Biomech..

[14] Beidokhti HN, Janssen D, van de Groes S, Hazrati J, Van den Boogaard T, Verdonschot N (2017). The influence of ligament modelling strategies on the predictive capability of finite element models of the human knee joint. J. Biomech..

[15] Eskelinen AS, Tanska P, Florea C, Orozco GA, Julkunen P, Grodzinsky AJ, Korhonen RK (2020). Mechanobiological model for simulation of injured cartilage degradation via pro-inflammatory cytokines and mechanical stimulus. PLoS Comput. Biol..

[16] Guo H, Santner TJ, Lerner AL, Maher SA (2017). Reducing uncertainty when using knee-specific finite element models by assessing the effect of input parameters. J. Orthop. Res..

[17] Gustafson JA, Elias JJ, Debski RE, Farrokhi S (2019). Development and validation of a kinematically-driven discrete element model of the patellofemoral joint. J. Biomech..

[18] Mononen ME, Tanska P, Isaksson H, Korhonen RK (2016). A novel method to simulate the progression of collagen degeneration of cartilage in the knee: Data from the osteoarthritis initiative. Sci. Rep..

[19] Mallinos A, Jones K, Davis B (2022). Pivot shift and Lachman test simulation-based exploration in juvenile populations for accurately predicting anterior tibial translation. J. Biomech..

[20] Fabricant PD, Lakomkin N, Sugimoto D, Tepolt FA, Stracciolini A, Kocher MS (2016). Youth sports specialization and musculoskeletal injury: A systematic review of the literature. Phys. Sportsmed..

[21] Barzan M, Modenese L, Carty CP, Maine S, Stockton CA, Sancisi N, Lewis A, Grant J, Lloyd DG, da Luz SB (2019). Development and validation of subject-specific pediatric multibody knee kinematic models with ligamentous constraints. J. Biomech..

[22] Baldwin MA, Clary CW, Fitzpatrick CK, Deacy JS, Maletsky LP, Rullkoetter PJ (2012). Dynamic finite element knee simulation for evaluation of knee replacement mechanics. J. Biomech..

[23] Blankevoort L, Huiskes R (1996). Validation of a three-dimensional model of the knee. J. Biomech..

[24] Park H-S, Ahn C, Fung DT, Ren Y, Zhang L-Q (2010). A knee-specific finite element analysis of the human anterior cruciate ligament impingement against the femoral intercondylar notch. J. Biomech..

[25] Song Y, Debski RE, Musahl V, Thomas M, Woo SL-Y (2004). A three-dimensional finite element model of the human anterior cruciate ligament: A computational analysis with experimental validation. J. Biomech..

[26] Xie, F., Yang, L., Guo, L., Wang, Z. -J. & Dai, G. A study on construction three-dimensional nonlinear finite element model and stress distribution analysis of anterior cruciate ligament (2009). 10.1115/1.4000167 20524730

[27] Abdel-Rahman EM, Hefzy MS (1998). Three-dimensional dynamic behaviour of the human knee joint under impact loading. Med. Eng. Phys..

[28] Haut Donahue TL, Hull M, Rashid MM, Jacobs CR (2002). A finite element model of the human knee joint for the study of tibio-femoral contact. J. Biomech. Eng..

[29] Pena E, Martinez M, Calvo B, Palanca D, Doblaré M (2005). A finite element simulation of the effect of graft stiffness and graft tensioning in ACL reconstruction. Clin. Biomech..

[30] Ramaniraka N, Saunier P, Siegrist O, Pioletti DP (2007). Biomechanical evaluation of intra-articular and extra-articular procedures in anterior cruciate ligament reconstruction: A finite element analysis. Clin. Biomech..

[31] Schache AG, Baker R (2007). On the expression of joint moments during gait. Gait Posture.

[32] Hermens HJ, Freriks B, Disselhorst-Klug C, Rau G (2000). Development of recommendations for SEMG sensors and sensor placement procedures. J. Electromyogr. Kinesiol..

[33] Rajagopal A, Dembia CL, DeMers MS, Delp DD, Hicks JL, Delp SL (2016). Full-body musculoskeletal model for muscle-driven simulation of human gait. IEEE Trans. Biomed. Eng..

[34] Delp SL, Anderson FC, Arnold AS, Loan P, Habib A, John CT, Guendelman E, Thelen DG (2007). OpenSim: Open-source software to create and analyze dynamic simulations of movement. IEEE Trans. Biomed. Eng..

[35] Kainz H, Hoang HX, Stockton C, Boyd RR, Lloyd DG, Carty CP (2017). Accuracy and reliability of marker-based approaches to scale the pelvis, thigh, and shank segments in musculoskeletal models. J. Appl. Biomech..

[36] Modenese L, Ceseracciu E, Reggiani M, Lloyd DG (2016). Estimation of musculotendon parameters for scaled and subject specific musculoskeletal models using an optimization technique. J. Biomech..

[37] Handsfield GG, Meyer CH, Hart JM, Abel MF, Blemker SS (2014). Relationships of 35 lower limb muscles to height and body mass quantified using MRI. J. Biomech..

[38] Pizzolato C, Lloyd DG, Sartori M, Ceseracciu E, Besier TF, Fregly BJ, Reggiani M (2015). CEINMS: A toolbox to investigate the influence of different neural control solutions on the prediction of muscle excitation and joint moments during dynamic motor tasks. J. Biomech..

[39] Sartori M, Farina D, Lloyd DG (2014). Hybrid neuromusculoskeletal modeling to best track joint moments using a balance between muscle excitations derived from electromyograms and optimization. J. Biomech..

[40] Esrafilian A, Stenroth L, Mononen ME, Vartiainen P, Tanska P, Karjalainen PA, Suomalainen J-S, Arokoski JP, Saxby DJ, Lloyd DG (2022). An EMG-assisted muscle-force driven finite element analysis pipeline to investigate joint-and tissue-level mechanical responses in functional activities: Towards a rapid assessment toolbox. IEEE Trans. Biomed. Eng..

[41] Halonen KS, Mononen M, Jurvelin J, Töyräs J, Kłodowski A, Kulmala J-P, Korhonen R (2016). Importance of patella, quadriceps forces, and depthwise cartilage structure on knee joint motion and cartilage response during gait. J. Biomech. Eng..

[42] Blankevoort, L. & Huiskes, R. Ligament-bone interaction in a three-dimensional model of the knee (1991). 10.1115/1.2894883 1921352

[43] Smith CR, Brandon SC, Thelen DG (2019). Can altered neuromuscular coordination restore soft tissue loading patterns in anterior cruciate ligament and menisci deficient knees during walking?. J. Biomech..

[44] Bolcos PO, Mononen ME, Mohammadi A, Ebrahimi M, Tanaka MS, Samaan MA, Souza RB, Li X, Suomalainen J-S, Jurvelin JS (2018). Comparison between kinetic and kinetic-kinematic driven knee joint finite element models. Sci. Rep..

[45] Tanska P, Mononen ME, Korhonen RK (2015). A multi-scale finite element model for investigation of chondrocyte mechanics in normal and medial meniscectomy human knee joint during walking. J. Biomech..

[46] Villegas DF, Maes JA, Magee SD, Donahue TLH (2007). Failure properties and strain distribution analysis of meniscal attachments. J. Biomech..

[47] Klets O, Mononen ME, Tanska P, Nieminen MT, Korhonen RK, Saarakkala S (2016). Comparison of different material models of articular cartilage in 3D computational modeling of the knee: Data from the osteoarthritis initiative (OAI). J. Biomech..

[48] Ottoboni A, Parenti-Castelli V, Sancisi N, Belvedere C, Leardini A (2010). Articular surface approximation in equivalent spatial parallel mechanism models of the human knee joint: An experiment-based assessment. Proc. Inst. Mech. Eng. Part H J. Eng. Med..

[49] Sancisi N, Parenti-Castelli V (2011). A novel 3D parallel mechanism for the passive motion simulation of the patella-femur-tibia complex. Meccanica.

[50] Sancisi, N. & Parenti-Castelli, V. A new kinematic model of the passive motion of the knee inclusive of the patella (2011).

[51] Benoit DL, Ramsey DK, Lamontagne M, Xu L, Wretenberg P, Renström P (2006). Effect of skin movement artifact on knee kinematics during gait and cutting motions measured in vivo. Gait Posture.

[52] Gilbert S, Chen T, Hutchinson ID, Choi D, Voigt C, Warren RF, Maher SA (2014). Dynamic contact mechanics on the tibial plateau of the human knee during activities of daily living. J. Biomech..

[53] Kefala V, Cyr AJ, Harris MD, Hume DR, Davidson BS, Kim RH, Shelburne KB (2017). Assessment of knee kinematics in older adults using high-speed stereo radiography. Med. Sci. Sports Exerc..

[54] Kozanek M, Hosseini A, Liu F, Van de Velde SK, Gill TJ, Rubash HE, Li G (2009). Tibiofemoral kinematics and condylar motion during the stance phase of gait. J. Biomech..

[55] Gray HA, Guan S, Pandy MG (2017). Accuracy of mobile biplane X-ray imaging in measuring 6-degree-of-freedom patellofemoral kinematics during overground gait. J. Biomech..

[56] Thomeer LT, Lin Y-C, Pandy MG (2020). Load distribution at the patellofemoral joint during walking. Ann. Biomed. Eng..

[57] Orozco GA, Tanska P, Mononen ME, Halonen KS, Korhonen RK (2018). The effect of constitutive representations and structural constituents of ligaments on knee joint mechanics. Sci. Rep..

