# Supplementary material for: Validation and evaluation of subject-specific finite element models of the pediatric knee
Source: Sci Rep. 2023 Oct 26;13:18328. doi: 10.1038/s41598-023-45408-5 (PMC10603053; doi:10.1038/s41598-023-45408-5)
Supplement: Supplementary file 1 — Supplementary Information. [file 41598_2023_45408_MOESM1_ESM.docx]

**Supplementary material**

**Validation and evaluation of subject-specific finite element models of the pediatric knee**

Ayda Karimi Dastgerdi^1*^, Amir Esrafilian^2^, Christopher P. Carty^1,3^, Azadeh Nasseri^1^, Alireza Yahyaiee Bavil^1^, Martina Barzan^1^, Rami K. Korhonen^2^, Ivan Astori^3^, Wayne Hall^4^, and David John Saxby^1^

^1^Griffith Centre of Biomedical and Rehabilitation Engineering (GCORE), Menzies Health Institute Queensland and the Advanced Design and Prototyping Technologies Institute (ADAPT), Griffith University, Gold Coast, QLD, Australia.

^*^email: ayda.karimidastgerdi@griffithuni.edu.au

^2^Department of Technical Physics, University of Eastern Finland, Kuopio, Finland.

^3^Department of Orthopedics, Children’s Health Queensland Hospital and Health Service, QLD, Australia.

^4^School of Engineering and Built Environment - Mechanical Engineering and Industrial Design, Griffith University, Gold Coast, QLD, Australia.

1. Muscle activation

A multi degree-of-freedom(multi-DoFs) calibration [1, 2] procedure was executed to fine-tune the neuromuscular parameters pertaining to all musculature in the right lower limb within the context of the musculoskeletal (MSK) model for each subject. This calibration encompassed five degrees of freedom, comprising the hip (with three DoF), the knee (with one DoF), and the ankle (with one DoF). The key neuromuscular parameters addressed included the maximum isometric force, tendon slack-length, optimal fiber-length, EMG-to-activation recursive filter coefficients, and the nonlinear shape-factor [3, 4].

Within the calibration, the above-mentioned neuromuscular parameters were adjusted in order to minimize the following cost function ($f_{\mathrm{cost}}^{\mathrm{cal}}$) [1]:

$f_{\mathrm{cost}}^{\mathrm{cal}}=\sum_{t=1}^{N_{\mathrm{trials}}} \sum_{d=1}^{N_{\mathrm{DoFs}}} [\frac{1}{N_{\mathrm{rows}}}\sum_{r=1}^{N_{\mathrm{rows}}} [\frac{{[M_{t,d,r}^{\exp}-M_{t,d,r}]}^{2}}{variance(M_{t,d,r}^{\exp})}+p_{r}]]$ (Eq.1)

where $N_{\mathrm{trials}}$ is number of trials used for calibration, $N_{\mathrm{DoFs}}$ is the number of DoFs, $N_{\mathrm{rows}}$ is the number of data points in the trial, $M_{t,d,r}^{\exp}$ is the experimental moment, $M_{t,d,r}$ is the moments estimated by the CEINMS, and $p_{r}$ is a penalty factor to discourage the non-physiological solutions, i.e., muscle parameters [1]. The $M_{t,d,r}$ in Eq.1 is calculated using the estimated muscle forces by the execution step of the CEINMS, in which the following cost function ($f_{\mathrm{cost}}^{exc,t}$) is minimized:

$f_{\mathrm{cost}}^{exc,t}=\alpha\sum_{d=1}^{N_{\mathrm{DoFs}}} \sum_{r=1}^{N_{\mathrm{rows}}} \left[ M_{t,d,r}^{\exp}-M_{t,d,r} \right]^{2}+\beta\sum_{j=1}^{N_{\mathrm{muscles}}} [\sum_{r=1}^{N_{\mathrm{rows}}} {e_{t,j,r}}^{2}]+ \gamma\sum_{k=1}^{N_{\mathrm{EMGs}}} [\sum_{r=1}^{N_{\mathrm{rows}}} {[e_{t,k,r}^{\exp}-e_{t,k,r}]}^{2}]$ (Eq. 2)

where $e_{t,j,r}$ is the j^th^ muscle excitation, $e_{t,k,r}^{\exp}$ is the EMG envelope measured from k^th^ muscle, and $e_{t,k,r}$ is the estimated muscle excitation of the k^th^ measured muscle. The weight factors $\alpha$, $\beta$, and $\gamma$ are positive weighting coefficients to alter the weight of each term [5].

The muscle activation of some knee extensors (i.e., vastus lateralis, rectus femoris, and vastus medialis) and knee flexors (i.e., biceps femoris, gastrocnemius lateralis, gastrocnemius medialis, and semitendinosus), estimated by CEINMS, were averaged across the eight subjects (i.e., TD 1-8) and compared with published data from literature [6-8].

Figures 1 and 2 depict the muscle activation profiles of knee flexors and extensors in comparison to previously published data [6-8]. In Figure 1 and 2, the shaded regions in green represent values from pediatric literature for major muscles and muscle groups, aligning with our collected data [6]. It is noteworthy that literature lacks reports on activation patterns of deep or unmeasured muscles in typically developing children during walking. The activation patterns displayed in Figures 1 and 2 are consistent with adult literature data, as evidenced by the similarity in patterns and the occurrence of peak activations. However, disparities in activation values between our study and the literature may arise from the fact that our subjects comprise a pediatric population, whereas benchmark data from the literature predominantly pertains to adults. Yet, the agreement in patterns and peak occurrences between our data and the literature affirms the accurate synthesis of muscle excitations, regardless of whether they are derived from direct EMG measurements or estimated through specific analytical criteria. Furthermore, it is crucial to acknowledge the substantial variability in literature-derived values, reflecting the highly subject-specific nature of observed activation patterns and magnitudes. This consideration underpins our rationale for employing CEINMS-based modeling over mechanical optimization when determining neural solutions.


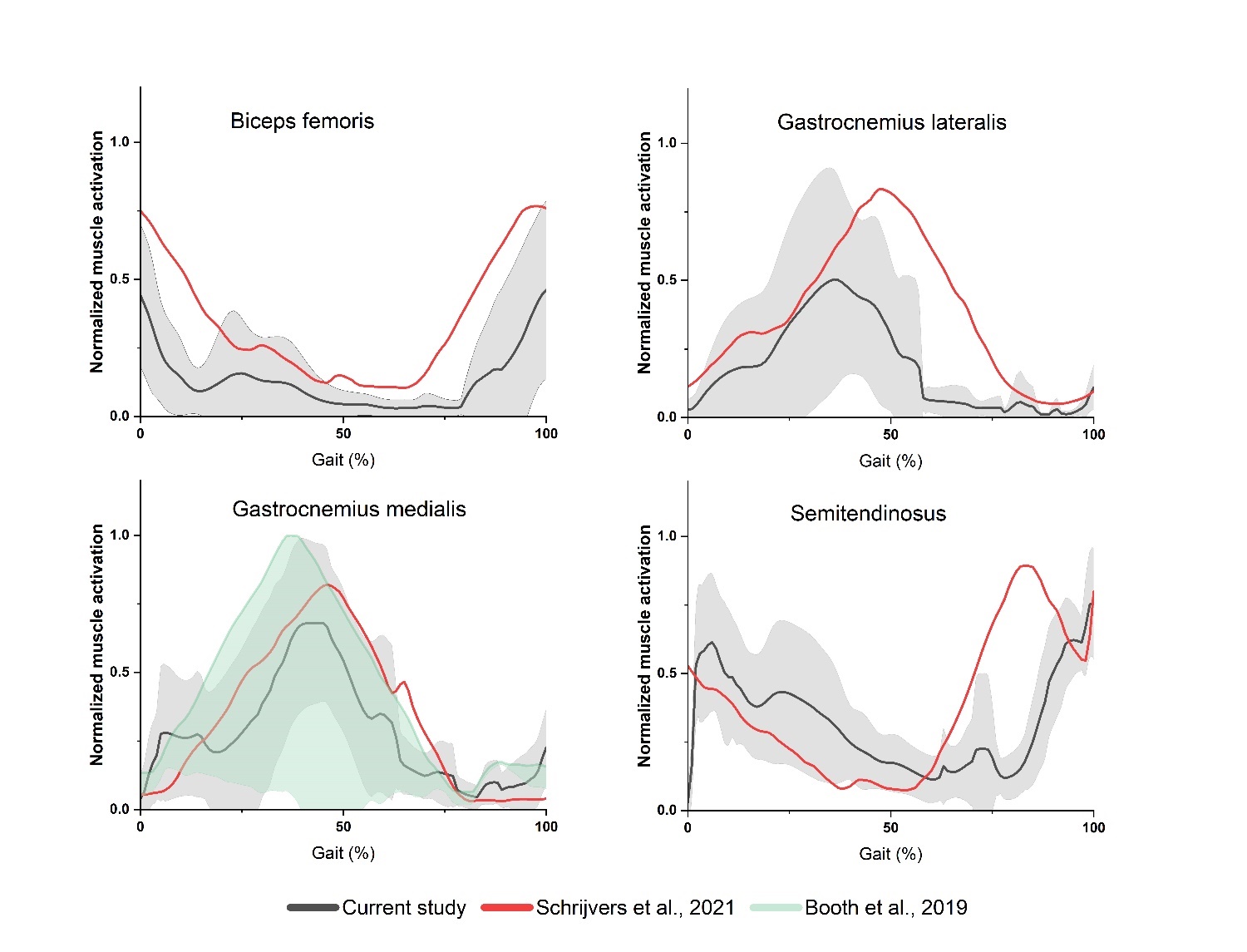


Figure S1: Muscle activation patterns across eight subjects (mean ± SD) of knee flexors (i.e., grey area) compared to published data (i.e., green area and red line) [6, 7].


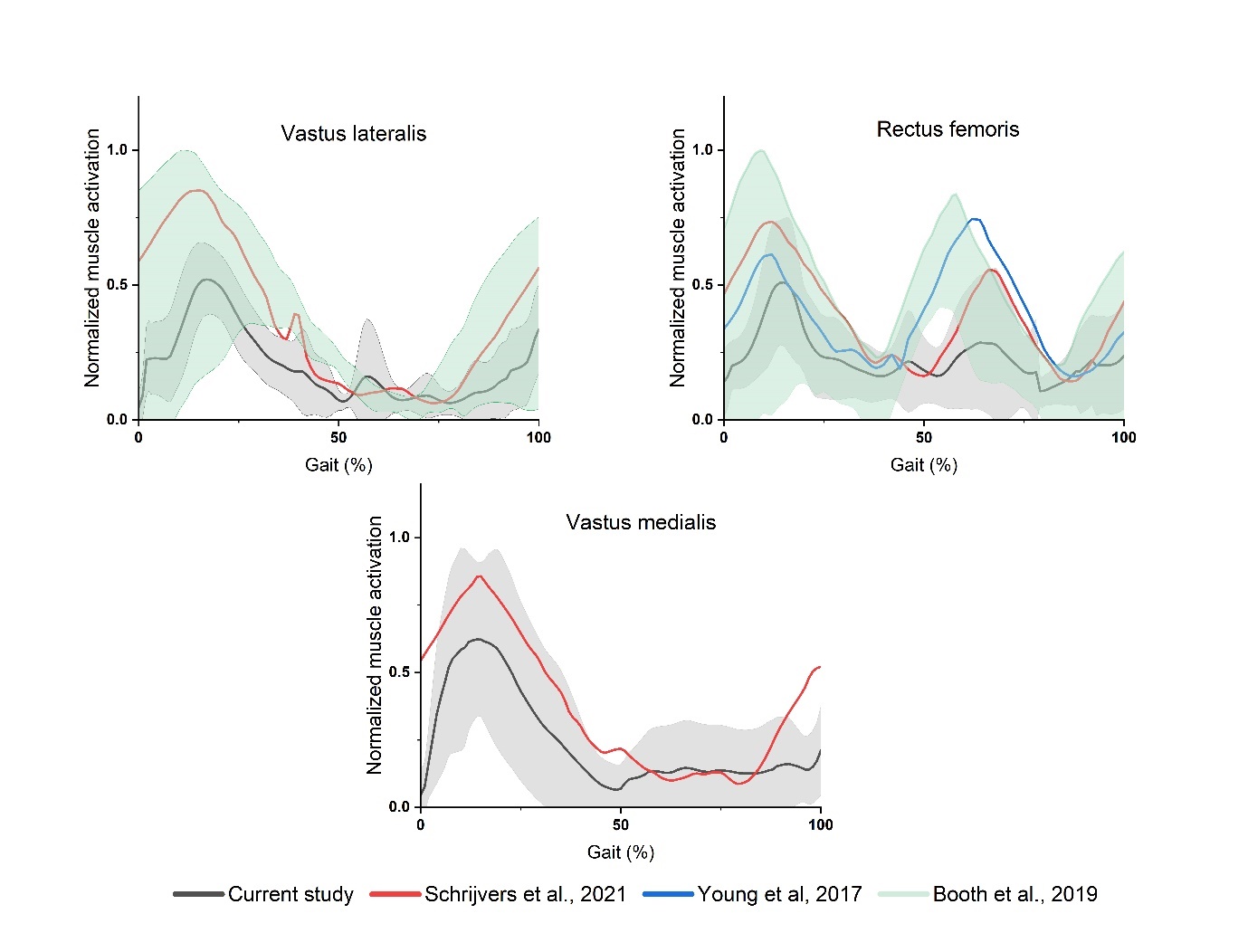


Figure S2: Muscle activation patterns across eight subjects (mean ± SD) of knee extensors compared to published data (i.e., green area, red, and blue lines) [6-8].

# Atlas-based approach


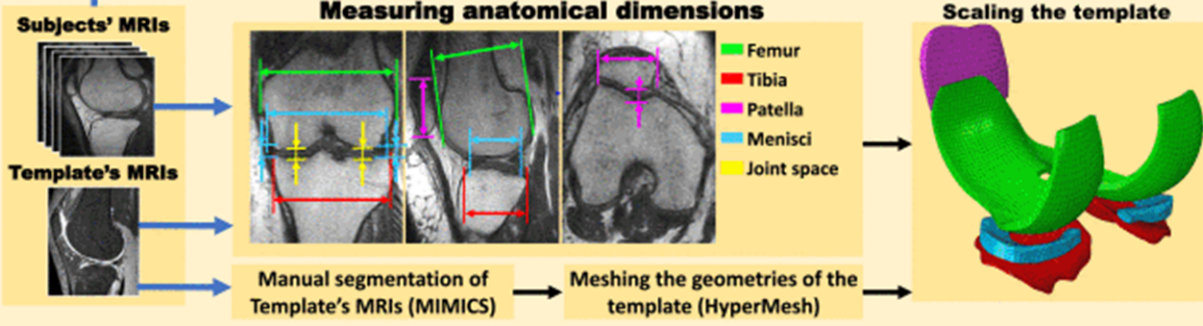


Figure S3: Measurements of anatomical dimensions conducted in an atlas-based approach were employed to develop subject-specific Finite Element models for this study by anisotropically scaling a template model.

# Loading and boundary conditions


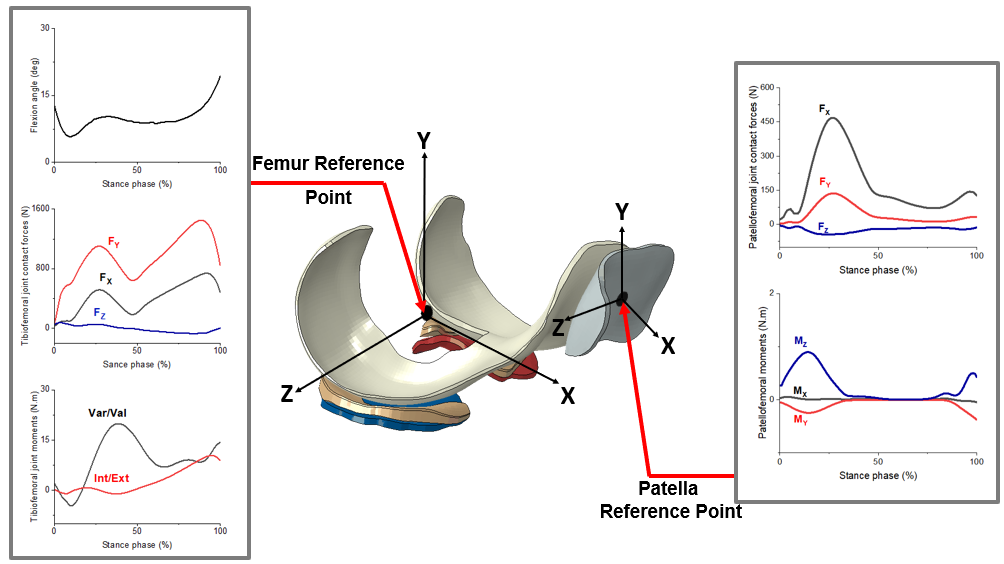


Figure S4: The application of loading and boundary conditions to the Finite Element models. Specifically, the tibiofemoral (TFJ) flexion angle (obtained from Inverse Kinematics analysis), along with knee internal/external and varus/valgus moments (comprising contributions from Inverse Dynamics, in addition to moments generated by muscles in abduction/adduction and internal/external Degrees of Freedom), as well as tibiofemoral contact forces (in the three-dimensional XYZ coordinate system), were applied to the femoral reference point. Similarly, patellar flexion/extension, varus/valgus, and internal/external moments originating from the quadriceps, in addition to patellofemoral contact forces (in the three-dimensional XYZ coordinate system), were applied at the patellar reference point.

**References**

[1] C. Pizzolato, D. G. Lloyd, M. Sartori, E. Ceseracciu, T. F. Besier, B. J. Fregly, and M. Reggiani, “CEINMS: A toolbox to investigate the influence of different neural control solutions on the prediction of muscle excitation and joint moments during dynamic motor tasks,” *J. Biomech.,* vol. 48, no. 14, pp. 3929-3936, 2015.

[2] M. Sartori, M. Reggiani, D. Farina, and D. G. Lloyd, “EMG-driven forward-dynamic estimation of muscle force and joint moment about multiple degrees of freedom in the human lower extremity,” *PLoS One,* vol. 7, no. 12, pp. e52618, 2012.

[3] T. S. Buchanan, D. G. Lloyd, K. Manal, and T. F. Besier, “Neuromusculoskeletal modeling: estimation of muscle forces and joint moments and movements from measurements of neural command,” *J. Appl. Biomech.,* vol. 20, no. 4, pp. 367-395, 2004.

[4] D. G. Lloyd, and T. F. Besier, “An EMG-driven musculoskeletal model to estimate muscle forces and knee joint moments in vivo,” *J. Biomech.,* vol. 36, no. 6, pp. 765-776, 2003.

[5] M. Sartori, D. Farina, and D. G. Lloyd, “Hybrid neuromusculoskeletal modeling to best track joint moments using a balance between muscle excitations derived from electromyograms and optimization,” *J. Biomech.,* vol. 47, no. 15, pp. 3613-3621, 2014/11/28/, 2014.

[6] A. T. Booth, M. M. van der Krogt, J. Harlaar, N. Dominici, and A. I. Buizer, “Muscle synergies in response to biofeedback-driven gait adaptations in children with cerebral palsy,” *Front. Physiol.,* vol. 10, pp. 1208, 2019.

[7] J. C. Schrijvers, J. C. van den Noort, M. van der Esch, and J. Harlaar, “Responses in knee joint muscle activation patterns to different perturbations during gait in healthy subjects,” *J. Electromyogr. Kinesiol.,* vol. 60, pp. 102572, 2021.

[8] A. J. Young, H. Gannon, and D. P. Ferris, “A biomechanical comparison of proportional electromyography control to biological torque control using a powered hip exoskeleton,” *Front. bioeng. biotechnol.,* vol. 5, pp. 37, 2017.
